# A Retrospective Analysis Of Different Contingent Screening Models For Fetal Down Syndrome In Southwestern China

**DOI:** 10.1038/s41598-020-66320-2

**Published:** 2020-06-11

**Authors:** Wei Luo, Bin He, Daiwen Han, Lixing Yuan, Xinlian Chen, Ling Pang, Jun Tang, Fene Zou, Kai Zhao, Yepei Du, Hongqian Liu

**Affiliations:** 1 Department of Obstetrics and Gynecology, West China Second University Hospital of Sichuan University, Chengdu, 610041 China; 2 Key Laboratory of Birth Defects and Related Diseases of Women and Children, Sichuan University, Ministry of Education, Chengdu, 610041 China

**Keywords:** Biomarkers, Diseases, Medical research

## Abstract

To discuss combinations of traditional screening and noninvasive prenatal screening (NIPS) and to compare which traditional screening is the most suitable first-line screening approach to NIPS, pregnant women were recruited in this retrospective observational study. Pregnant women underwent one of four traditional screening tests. The 9 contingent models were combined by high risk cut-offs of 1:50, 1:100, 1:270 and intermediate risk cut-offs of 1:1000, 1:1500, 1:2000. We analyzed cost and performance of various screening models with contingent screening of different risk cut-offs. Compared with other screening tests, combined first-trimester screening (CFTS) had the lowest proportion of high risk (≥1:270) with the highest detection rate (DR) (78.79%) and the lowest proportion of intermediate risk (1:271~1:1000). When intermediate risk was 1:51 ~1:1500, CFTS as first-line screening had the lowest cost with DR of 93.94%. Other screening tests as the first-line screening with intermediate risk of 1:51~1:1000 had the lowest cost, there DR were 90.91%, 84.62%, 91.67%, respectively. Our study demonstrated if only one traditional screening was allowed to screen pregnant women, CFTS was recommended as the first choice. According to local health and economic conditions, adopting appropriate traditional screening with suitable cut-offs as first-line screening will contributed to a cost-effective screening model.

## Introduction

Down syndrome (DS), also known as trisomy 21 syndrome, is the most common chromosomal abnormality, with incidence of 1 in 1000 to 1 in 700 live births^1,2^. DS patients have characteristic facial features, such as wide eye distance, low bridge of nose, eyelid cleft and so on, which are often manifested as moderate to severe inherited intellectual disability and abnormal growth and development^2^. Down syndrome usually has multiple system complications^3^, causing serious mental and economic burden to the family and society. At present, there is no effective treatment for Down syndrome, which can effectively reduce the birth rate of Down syndrome. It is mainly to find pregnancies considered to be at high risk as determined by prenatal aneuploidy screening. Providing high risk pregnancies prenatal diagnosis, the analysis of fetus chromosome collected via amniocentesis or chorionic villus sampling^4,5^.

When serum biochemical screening was introduced in the China, a hybrid approach was implemented in which amniocentesis was offered to all patients ≥35 years old and to patients <35 years old who were at increased risk based on the maternal serum markers available at that time. Over time, the screening has evolved by using double screening, triple screening, quadruple screening, CFTS, integrated screening and sequential screening. The efficiency of screening tests are related to the screening strategies, which can vary with the number of biochemical makers, the timing of screening, cut-off value and positive detection rate that we set. Different countries and regions adopt different screening strategies.

In the past decade, NIPS based on massively parallel genomic sequencing (MPS) technology has been widely applied for the clinical detection of trisomy 21, trisomy 18, and trisomy 13^6^. A plenty of researches have indicated the excellent performance of NIPS in fetal Down syndrome screening^7–10^. While NIPS as first-line screening has been suggested, but it was too expensive to be popularized in developing countries. Recent studies show that a contingent strategy was more cost-effective, in which a high risk group is identified through traditional screening methods and only pregnancies in this group are offered NIPS screening^11–17^. Contingent screening has the potential to reduce invasive diagnostic test and to increase the detection rate of fetal Down syndrome, depending on the different traditional screening risk cut-offs^18–20^. In developing countries, there are only a few retrospective studies of large samples to explore the combinations of traditional screening and NIPS and to compare which strategy is the most suitable first-line traditional screening test approach to NIPS.

Complicating matters further, traditional screening tests used vary significantly in developing country, such as China. In this study, we evaluated various screening models with different cut-offs to determine the best strategy for implementing contingent screening, considering with their clinical performance and cost.

## Results

Pregnancy characteristics of women with known outcomes are summarized in Table 1. There were 33, 11, 13, 60 cases of trisomy 21 in combined first-trimester screening, quadruple screening, triple screening and double screening, respectively. The incidence of Down syndrome were 1/658,1/735,1/1053,1/1141 in combined first-trimester screening, quadruple screening, triple screening and double screening, respectively.

**Table 1:** Pregnancy characteristics of women with four traditional screening tests.

Characteristic	CFTS	quadruple screening	triple screening	double screening
**Number**	21713	8087	13692	68512
**Age distribution**				
Maternal age at expected date of delivery <35 years(%)	21358(98.37%)	7953(98.34%)	13500(98.6%)	67853(99.04%)
Maternal age at expected date of delivery ≥35 years(%)	355(1.63%)	134(1.66%)	192(1.4%)	659(0.96%)
Maternal age (years) at expected date of delivery (IQR)	29.91(27.97–29.91)	29.31(27.11–31.88)	26.55(23.97–29.2)	26.64(24.03–29.19)
**Method of conception (%)**				
Spontaneous	20594(94.85%)	7831(96.83%)	13651(99.7%)	68270(99.65%)
Assisted	1119(5.15%)	256(3.17%)	41(0.3%)	242(0.35%)
**Median maternal weight (IQR)**	53(49–58)	54.5(50–60)	53.5(49–59)	54(49–59.5)
**Median gestational age (days) at blood sample (IQR)**	85(85–91)	115(112–120)	122(116–129)	120(115–127)
**Insulin-dependent diabetes (%)**	14(0.06%)	6(0.07%)	8(0.06%)	42(0.06%)
**Smoker (%)**	160(0.74%)	56(0.69%)	25(0.18%)	69(0.1%)
**Incidence of Down syndrome(‰)**	33(1.52,1/658)	11(1.36,1/735)	13(0.95,1/1053)	60(0.88,1/1141)

*Data are given as median (interquartile range) or n (%). IQR, interquartile range.

### Performance of traditional screening

The number of patients who reached the three group of the risk based on the screening tests used is shown in Table 2. Following combined first-trimester screening, 370 (1.7%), 732 (3.37%) and 20611(94.92%) patients were classified as high risk, intermediate risk and low risk, respectively. Following quadruple screening, 306(3.78%), 598 (7.39%) and 7183(88.82%) patients were classified as high risk, intermediate risk and low risk, respectively. Following triple screening, 415(3.03%), 943(6.89%) and 12334(90.08%) patients were classified as high risk, intermediate risk and low risk, respectively. Following double screening, 3629 (5.3%), 7753 (11.32%) and 57130(83.39%) patients were classified as high risk, intermediate risk and low risk, respectively. Comparing the proportion of high, intermediate, low risk among the four screening tests, the difference is statistically significant (*P* < 0.01). The combined first-trimester screening had the lowest proportion of high risk and intermediate risk, followed by triple screening, quadruple screening and double screening.

**Table 2:** Performance of traditional screening.

Risk stratification	CFTS		quadruple screening		triple screening		double screening	
	n(%)	T21(%)	n(%)	T21(%)	n(%)	T21(%)	n(%)	T21(%)
high risk (risk ≥ 1/270)	370 (1.70%)	26 (78.79%)	306 (3.78%)	8 (72.73%)	415 (3.03%)	9 (69.23%)	3629 (5.30%)	42 (70.00%)
intermediate risk (1/1000 ≤ risk < 1/270)	732 (3.37%)	3 (9.09%)	598 (7.39%)	2 (18.18%)	943 (6.89%)	2 (15.38%)	7753 (11.32%)	13 (21.67%)
low risk (risk < 1/1000)	20611 (94.92%)	4 (12.12%)	7183 (88.82%)	1 (9.09%)	12334 (90.08%)	2 (15.38%)	57130 (83.39%)	5 (8.33%)

The high risk detection rate of combined first-trimester screening was 78.79% (26 out of 33 cases), while that for quadruple screening, triple screening and double screening was 72.73% (8 out of 11 cases), 69.23% (9 out of 13 cases) and 70.00% (42 out of 60 case), respectively. There was no significant difference in the detection rate between the four methods (*P* = 0.829). Table 2 and Table 3.

**Table 3:** Evaluation index of traditional screening.

index	CFTS	quadruple screening	triple screening	double screening
TPR(%)	78.79	72.73	69.23	70.00
TNR(%)	98.41	96.31	97.09	94.76
FPR(%)	1.59	3.69	2.91	5.24
PPV(%)	7.03	2.61	2.17	1.16

*TPR, true positive rate, also known as detection rate. TNR, true negative rate. FPR, false positive rate. PPV, positive predictive value.

With high risk cut-off of 1:270, the true negative rate of combined first-trimester screening, quadruple screening, triple screening and double screening were 98.41%(21336 out of 21680 cases), 96.31% (7778 out of 8076 cases), 97.09% (13543 out of 13949 cases), 94.76% (64865 out of 68452 cases), respectively. The false positive rates of combined first-trimester screening, quadruple screening, triple screening and double screening were 1.59%(344 out of 21680 cases), 3.69% (298 out of 8076 cases), 2.91% (406 out of 13949 cases), 5.24% (3587 out of 68452 cases), respectively. The positive predictive value of combined first-trimester screening, quadruple screening, triple screening and double screening were 7.03%(26 out of 370 cases), 2.61% (8 out of 306 cases), 2.17% (9 out of 415 cases), 1.16% (42 out of 3629 cases), respectively. And there were significant difference in the true negative rate, false positive rates and positive predictive value between the four methods (*P* < 0.01).

### Cost

Costs and diagnoses of the contingent models are shown in Table 4. When high risk cut-off was 1:50 and intermediate risk cut-off was 1:1000, the overall cost of quadruple screening, triple screening and double screening were all the lowest at their group, they were 892677USD, 1352854 USD, 6728175 USD, respectively. The overall cost of combined first-trimester screening was the lowest (1976253USD) when high risk cut-off was 1:50 and intermediate risk cut-off was 1:1500. For combined first-trimester screening, the cost of contingent models ranged from 1976253USD (detection rate 93.94%, intermediate risk 1:51~1:1500) to 2173069USD (detection rate 87.88%, intermediate risk 1:271~1:1000). For quadruple screening, the cost of contingent models ranged from 892677USD (intermediate risk 1:51~1:1000) to 1111788USD (intermediate risk 1:271~1:2000) at detection rate of 90.91%. For triple screening, the cost of contingent models ranged from 1352854USD (intermediate risk 1:51~1:1000) to 1693233USD (intermediate risk 1:271~1:2000) at detection rate of 84.62%. And for double screening, the cost of contingent models ranged from 6728175USD (detection rate 91.67%, intermediate risk 1:51~1:1000) to 9167493USD (detection rate 98.33%, intermediate risk 1:51~1:2000).

**Table 4:** Costs of the contingent models.

1/1000 ≤ intermediate risk < 1/50	cFTS		quadruple screening		triple screening		double screening	
	n or %	USD	n or %	USD	n or %	USD	n or %	USD
DR of T21(%)	87.88		90.91		84.62		91.67	
diagnosis	124	47740	63	24255	68	26180	590	227150
NIPS	983	362727	845	311805	1296	478224	10846	4002174
missed detection for T21	4	738460	1	184615	2	369230	5	923075
screening	21713	1020511	8087	372002	13692	479220	68512	1575776
overall cost		2169438		892677		1352854		6728175
average cost per DS detected		74808		89268		122987		122330
**1/1500** ≤ **intermediate risk** < **1/50**								
DR of T21(%)	93.94		90.91		84.62		91.67	
diagnosis	126	48510	65	25025	71	27335	612	235620
NIPS	1458	538002	1146	422874	1781	657189	15169	5597361
missed detection for T21	2	369230	1	184615	2	369230	3	553845
screening	21713	1020511	8087	372002	13692	479220	68512	1575776
overall cost		1976253		1004516		1532974		7962602
average cost per DS detected		63750		100452		139361		139695
**1/2000** ≤ **intermediate risk** < **1/50**								
DR of T21(%)	93.94		90.91		84.62		98.33	
diagnosis	128	49280	66	25410	73	28105	633	243705
NIPS	1898	700362	1426	526194	2200	811800	19413	7163397
missed detection for T21	2	369230	1	184615	2	369230	1	184615
screening	21713	1020511	8087	372002	13692	479220	68512	1575776
overall cost		2139383		1108221		1688355		9167493
average cost per DS detected		69012		110822		153487		155381
**1/1000** ≤ **intermediate risk** < **1/100**								
DR of T21(%)	87.88		90.91		84.62		91.67	
diagnosis	190	73150	129	49665	155	59675	1455	560175
NIPS	917	338373	779	287451	1209	446121	9977	3681513
missed detection for T21	4	738460	1	184615	2	369230	5	923075
screening	21713	1020511	8087	372002	13692	479220	68512	1575776
overall cost		2170494		893733		1354246		6740539
average cost per DS detected		74845		89373		123113		122555
**1/1500** ≤ **intermediate risk** < **1/100**								
DR of T21(%)	93.94		90.91		84.62		91.67	
diagnosis	192	73920	130	50050	157	60445	1477	568645
NIPS	1392	513648	1080	398520	1694	625086	14300	5276700
missed detection for T21	2	369230	1	184615	2	369230	3	553845
screening	21713	1020511	8087	372002	13692	479220	68512	1575776
overall cost		1977309		1005187		1533981		7974966
average cost per DS detected		63784		100519		139453		139912
**1/2000** ≤ **intermediate risk** < **1/100**								
DR of T21(%)	93.94		90.91		84.62		98.33	
diagnosis	194	74690	132	50820	160	61600	1496	575960
NIPS	1832	676008	1360	501840	2113	779697	18274	6743106
missed detection for T21	2	369230	1	184615	2	369230	1	184615
screening	21713	1020511	8087	372002	13692	479220	68512	1575776
overall cost		2140439		1109277		1689747		9079457
average cost per DS detected		69046		110928		153613		153889
**1/1000** ≤ **intermediate risk** **<** **1/270**								
DR of T21(%)	87.88		90.91		84.62		91.67	
diagnosis	374	143990	309	118965	420	161700	3668	1412180
NIPS	732	270108	598	220662	943	347967	7753	2860857
missed detection for T21	4	738460	1	184615	2	369230	5	923075
screening	21713	1020511	8087	372002	13692	479220	68512	1575776
overall cost		2173069		896244		1358117		6771888
average cost per DS detected		74933		89624		123465		123125
**1/1500** ≤ **intermediate risk** < **1/270**								
DR of T21(%)	93.94		90.91		84.62		91.67	
diagnosis	376	144760	310	119350	422	162470	3689	1420265
NIPS	1207	445383	899	331731	1428	526932	12076	4456044
missed detection for T21	2	369230	1	184615	2	369230	3	553845
screening	21713	1020511	8087	372002	13692	479220	68512	1575776
overall cost		1979884		1007698		1537852		8005930
average cost per DS detected		63867		100770		139805		140455
**1/2000** ≤ **intermediate risk** < **1/270**								
DR of T21(%)	93.94		90.91		84.62		98.33	
diagnosis	378	145530	312	120120	424	163240	3709	1427965
NIPS	1647	607743	1179	435051	1847	681543	16050	5922450
missed detection for T21	2	369230	1	184615	2	369230	1	184615
screening	21713	1020511	8087	372002	13692	479220	68512	1575776
overall cost		2143014		1111788		1693233		9110806
average cost per DS detected		69129		111179		153930		154420

*The screening results were calculated independently.

Accordingly, When high risk cut-off was 1:50 and intermediate risk cut-off was 1:1000, average cost per DS detected of quadruple screening, triple screening and double screening were all the lowest at their group, they were 89268USD, 122987USD, 122330USD, respectively. The average cost per DS detected of combined first-trimester screening was the lowest (63750USD) when high risk cut-off was 1:50 and intermediate risk cut-off was 1:1500. For combined first-trimester screening, the average cost per DS detected of contingent models ranged from 63750USD (detection rate 93.94%, intermediate risk 1:51~1:1500) to 74933USD (detection rate 87.88%, intermediate risk 1:271~1:1000). For quadruple screening, the average cost per DS detected of contingent models ranged from 89268USD (intermediate risk 1:51~1:1000) to 111179USD (intermediate risk 1:271~1:2000) at detection rate of 90.91%. For triple screening, the average cost per DS detected of contingent models ranged from 122987USD (intermediate risk 1:51~1:1000) to 153930USD (intermediate risk 1:271~1:2000) at detection rate of 84.62%. And for double screening, the average cost per DS detected of contingent models ranged from 122330USD (detection rate 91.67%, intermediate risk 1:51~1:1000) to 155381USD (detection rate 98.33%, intermediate risk 1:51~1:2000).

## Discussion

Our study presents that because of the lowest proportion (1.7%) of high risk (cut-off 1:270) with the highest detection rate (78.79%) and the highest positive predictive value (7.03%), the performance of combined first-trimester screening is better than second trimester screening tests. Offering combined first-trimester screening led to the lowest number of invasive tests compared with other traditional screenings. And this consequence is consistent with Lan’s research report^21^. It is also an advantageous to offer combined first-trimester screening to reduce the risk of iatrogenic fetal loss. Influenced by economic situation, educational background and regional health level, pregnant women have low compliance with the second screening in integrated screening or sequential screening and most pregnant women have received only one traditional screening test in China. If only one traditional screening test is allowed to screen pregnant women regardless of first trimester or second trimester, combined first-trimester screening is recommended as the first choice.

According to Technical standards of prenatal screening and diagnosis for fetal common chromosomal abnormalities and open neural tube defects Part1 Maternal serum prenatal screening in second trimester (the Health Standards of the People’s Republic of China, 2010) and Technical Specification for Prenatal Screening and Diagnosis of NIPS (National Health Commission of the People’s Republic of China, 2016), women with advanced maternal age were offered the options of amniocentesis and other women received traditional screening as the first-line tests. According to the results of first-line screening, women with the high risk (≥1:270) were offered the options of amniocentesis, women with an intermediate risk (1:1000~1:271) were offered NIPS and those with a low risk (1:1000) were reassured that fetal trisomies were unlikely and no further testing was necessary. Using this strategy, our study shows contingent model that NIPS contingent on result of combined first-trimester screening has a higher detection rate compared with the second trimester screening tests. And this contingent model can reduce subsequent the number of NIPS, thus reducing the cost of health economics.

The cut-off value of traditional screening needs to consider detection rate, invasive prenatal diagnosis rate and health economic costs and cut-off values of traditional screening in different countries and regions are different. However, with the contingent strategy of traditional screening and NIPS, a more appropriate cut-off should be found. In a study by Gil *et al*., the authors suggested that NIPS could be offered as a contingent screen following combined first trimester screening results. They proposed that women with intermediate risk (defined as 1:11–2500 in that study) could also be offered NIPS^22^. Some researchers suggested that the introduction of NIPS as a second line screening test, conditional to a risk ≥1:1000 from Standard of Care screening, showed a 3% increase in the detection of trisomies, with a 71% decrease in the number of invasive tests performed^23^. Other previous study suggest that the use of risk scores between 1:251 and 1:1000 may be a more cost-effective threshold^24^. While international experience provides some insights, it is very difficult to forecast how the availability and accessibility of NIPS will affect screening for fetal Down syndrome in China, particularly across models with varied definitions of high and intermediate risk.

When adopting quadruple screening or triple screening or double screening as the first-line screening, with high risk cut-off was 1:50 and intermediate risk cut-off was 1:1000, the proportion of women receiving prenatal diagnosis and NIPS were smaller than other cut-off models. For the second trimester screening tests, after adjusting the different risks, with the increase number of invasive diagnostic test and NIPS, the detection rate increases with the increase of cost. The overall cost and average cost per DS detected of combined first-trimester screening as a first-line screening were both the lowest when intermediate risk defined as 1:51~1500. Moreover, the detection rate of 93.94% remained higher than other cut-off models of combined first-trimester screening. Using this intermediate risk of combined first-trimester screening, the number of missed cases, the number of prenatal diagnosis and NIPS were relatively lower.

The cost of these models is sensitive to variation in uptake in invasive testing and NIPS. The high risk cut-off and intermediate risk cut-off used in the contingent models both had an impact on overall cost and effectiveness. It is helpful to compare economic evaluations on different contingent screening models, costs and assumptions. Consequently, our study found that contingent screening with different cut-offs could offer more cost-effective models, which is consistent with international studies in Belgium, the Netherlands and the UK^25–27^. In line with the Italian report, we consider that contingent screening using conventional CFTS and second trimester screening tests is effective^28^. The cost of each policy as a function of Down syndrome case diagnosed seems the best criterion to substantiate this hypothesis: CFTS is the best policy for the selection of patients to provide NIPS.

## Conclusions

We describe the performance of contingent screening models using a range of increasingly sensitive traditional screening risk cut-offs from the retrospective of the Southwestern China public health system. Our study demonstrated if only one traditional screening test is allowed to screen pregnant women, combined first-trimester screening is recommended as the first choice. The combined first-trimester screening can reduce subsequent the number of NIPS, thus reducing the cost of health economics. The findings of the present study confirm that NIPS contingent on the results of combined first-trimester screening with intermediate risk of 1:51~1:1500 is a cost-effective means of screening model for fetal Down syndrome in Chinese populations.

To the best of our knowledge, the present study is the first in China to assess the combinations of traditional screening and NIPS and has compared which traditional screening using an appropriate intermediate risk the most suitable first-line screening test approach to NIPS. In view of the fact that the whole nation couldn’t be covered by only one screening method because of the different detection ability and economic situation among different regions of China, our study offer clinicians clues to take a right choice.

## Limitation

This study was limited to a public health system perspective across the duration of strategies of screening and diagnosis for fetal Down syndrome. The incidence of Down syndrome was biased among the groups, because an unknown proportion of fetuses might miscarry without fetal tissue diagnosis.

## Materials and Methods

### Study design

**Figure 1:**
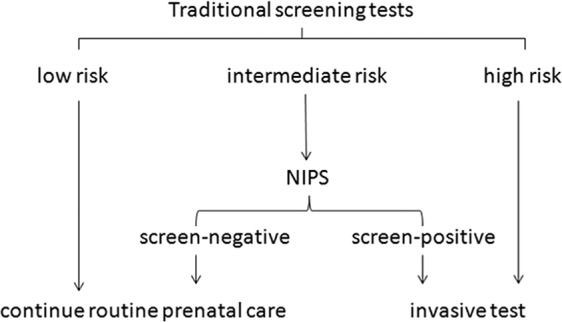
Contingent screening model.

This was a retrospective analysis of singleton pregnant women underwent combined first-trimester screening and quadruple screening, triple screening, double screening of second trimester. We collected and analyzed the results of combined first-trimester screening and the three screening tests of second trimester and there pregnancy outcomes, then we analyzed the performance of the four traditional screening tests. On this basis, it was assumed that the detection rate and false positive rate of NIPS were consistent with those reported, that meant detection rate of 99.5% and false positive of 0.5%^4^. The uptake rates of NIPS and invasive prenatal diagnosis were assumed to be 100%. The 9 contingent models were combined by high risk cut-offs of 1:50, 1:100, 1:270 and intermediate risk cut-offs of 1:1000, 1:1500 and 1:2000. And then, analyzed the cost and performance of various screening models when adopted to contingent screening model at different cut-offs, Fig. 1.

This study was conducted at Prenatal Diagnosis Center of West China Second University Hospital from January 2011 to December 2017. The study has been approved by the Institutional Ethics Committee of Sichuan University and all participants signed written informed consent prior to the test. The research was conducted in accordance with the relevant guidelines and clinical norms^29,30^, the details were as flows. The inclusion criteria were a maternal age of 16 years or older, pregnancy with a singleton live fetus, and the gestational age of 11 weeks through 13 weeks 6 days in the first trimester, 15 weeks through 20 weeks 6 days in the second trimester. Women were excluded from the study if they had a family history of chromosomal abnormalities or congenital malformations, medical and surgical diseases during pregnancy, twins or multiple pregnancies, and one of the twins disappearing. Fetal chromosome status was determined by amniocentesis; or by tissue sampling in cases of spontaneous pregnancy loss, pregnancy termination, or stillbirth; or by telephone follow-up results of pregnant women who did not receive amniocentesis^30^. The pregnant women were followed up by telephone six months after the pre delivery period to inquire about the pregnancy outcome and whether the fetus or newborn was normal^30^, cross-linked with the Sichuan Prenatal Diagnosis Information Network.

### Traditional screening

The combined first-trimester screening risk was calculated from measurements of nuchal translucency and two serum markers, pregnancy-associated plasma protein A (PAPP-A) and the free beta subunit of human chorionic gonadotropin (fβhCG), together with maternal age. The second trimester risk of quadruple screening was calculated from measurements of serum alpha-fetoprotein(AFP), the free beta subunit of human chorionic gonadotropin (fβhCG), unconjugated estriol and inhibin A, together with maternal age. The second trimester risk of triple screening was calculated from measurements of serum alpha-fetoprotein(AFP), the free beta subunit of human chorionic gonadotropin (fβhCG) and inhibin A, together with maternal age. The second-trimester risk of double screening was calculated from measurements of serum alpha-fetoprotein(AFP) and the free beta subunit of human chorionic gonadotropin (fβhCG), together with maternal age^31^.

Measurements of biochemical markers were converted into multiples of the median (MoM) for gestational age, adjusted for maternal weight, insulin-dependent diabetes mellitus, status of smoking and race. Biochemical markers MoM values were center-specific. The risk of fetal Down syndrome was estimated by multiplying the maternal age-specific odds of the live birth of an infant affected by Down syndrome by the likelihood ratio obtained from the overlapping Gaussian distributions of affected and unaffected pregnancies.

Detection of serum alpha-fetoprotein (AFP), the free beta subunit of human chorionic gonadotropin (fbhCG), unconjugated estriol and pregnancy-associated plasma protein A(PAPP-A) by using reagents and instruments of Perkin Elmer (USA). Detection of inhibin A by using reagents and instruments of Beckman Coulter (USA). Using Lifecycle (Perkin Elmer, USA) to calculate risk of combined first-trimester screening and double screening and use Prenatal Screening Software (TCSoft,China) to calculate risk of quadruple screening and triple screening.

### Cost of health service

A cost-effectiveness analysis was designed to evaluate contingent screening model in a retrospective traditional screening tests. In this contingent strategy, the follow-up to high risk further screening using amniocentesis and the follow-up to intermediate risk further screening using NIPS. As a result, the capability of intermediate risk will be reduced by 0.5% since the detection rate of NIPS screening is not 100% but rather 99.5%^4^. In China, the price of health services was obtained based on the charges set by the local government. This study treated the price of health service as cost because it was paid by the medical insurance and pregnant women. The cost comes from actual charge price and references^32–34^.

Direct medical costs included the costs of NIPS, traditional screening tests, amniocentesis, and social costs of missed detection for DS. Direct nonmedical costs and non-direct costs were not included in this study. Costs in Chinese yuan were converted into USD at the average of 2015–2017 exchange rate of 6.50 yuan= USD 1.00, Table 5.

**Table 5:** The cost of health service.

Items	Cost(USD)
CFTS^a^	47
double screening	23
triple screening	35
quadruple screening	46
NIPS	369
invasive diagnostic test^b^	385
missed detection for DS	184615

^a^The cost of combined first-trimester screening included measurements of PAPPA, freeβhCG and nuchal translucency; ^b^The cost of invasive diagnostic test included amniocentesis and analysis of fetal karyotype.

### Statistical analysis

Descriptive data were presented as median (interquartile range (IQR)) for continuous variables and as n (%) for categorical variables. Comparisons between groups were performed using Kruskal-Wallis H test or Fisher’s exact test for categorical variables. The statistical software package SPSS 23.0 (SPSS Inc., Chicago, IL, USA) was used for data analyses.
